# Patterns of multimorbidity in India: A nationally representative cross-sectional study of individuals aged 15 to 49 years

**DOI:** 10.1371/journal.pgph.0000587

**Published:** 2022-08-17

**Authors:** Jonas Prenissl, Jan-Walter De Neve, Nikkil Sudharsanan, Jennifer Manne-Goehler, Viswanathan Mohan, Ashish Awasthi, Dorairaj Prabhakaran, Ambuj Roy, Nikhil Tandon, Justine I. Davies, Rifat Atun, Till Bärnighausen, Lindsay M. Jaacks, Sebastian Vollmer, Pascal Geldsetzer

**Affiliations:** 1 Heidelberg Institute of Global Health, Medical Faculty and University Hospital, University of Heidelberg, Heidelberg, Germany; 2 Medical Faculty Heidelberg, Heidelberg University, Heidelberg, Germany; 3 Technical University of Munich, Munich, Germany; 4 Division of Infectious Diseases, Massachusetts General Hospital, Boston, MA, United States of America; 5 Madras Diabetes Research Foundation, ICMR Centre for Advanced Research on Diabetes, Chennai, Tamil Nadu, India; 6 Dr. Mohan’s Diabetes Specialities Centre,Chennai, Tamil Nadu, India; 7 Centre for Chronic Conditions and Injuries, Public Health Foundation of India, Gurugram, Haryana, India; 8 London School of Hygiene and Tropical Medicine, University of London, London, United Kingdom; 9 Department of Cardiology, Cardiothoracic Centre, All India Institute of Medical Sciences, New Delhi, India; 10 Department of Endocrinology and Metabolism, All India Institute of Medical Sciences, New Delhi, India; 11 Institute of Applied Health Research, Birmingham University, Birmingham, United Kingdom; 12 MRC/Wits Rural Public Health and Health Transitions Research Unit (Agincourt), School of Public Health, Faculty of Health Sciences, University of the Witwatersrand, Johannesburg, South Africa; 13 Department of Global Health and Population, Harvard T.H. Chan School of Public Health, Boston, MA, United States of America; 14 Harvard Medical School, Harvard University, Boston, MA, United States of America; 15 Africa Health Research Institute, Mtubatuba, KwaZulu-Natal, South Africa; 16 The Global Academy of Agriculture and Food Security, The University of Edinburgh, Midlothian, United Kingdom; 17 Department of Economics & Centre for Modern Indian Studies, University of Goettingen, Göttingen, Germany; 18 Division of Primary Care and Population Health, Department of Medicine, Stanford University, Stanford, CA, United States of America; 19 Chan Zuckerberg Biohub, San Francisco, CA, United States of America; Chiang Mai University, THAILAND

## Abstract

There is a dearth of evidence on the epidemiology of multimorbidity in low- and middle-income countries. This study aimed to determine the prevalence of multimorbidity in India and its variation among states and population groups. We analyzed data from a nationally representative household survey conducted in 2015–2016 among individuals aged 15 to 49 years. Multimorbidity was defined as having two or more conditions out of five common chronic morbidities in India: anemia, asthma, diabetes, hypertension, and obesity. We disaggregated multimorbidity prevalence by condition, state, rural versus urban areas, district-level wealth, and individual-level sociodemographic characteristics. 712,822 individuals were included in the analysis. The prevalence of multimorbidity was 7·2% (95% CI, 7·1% - 7·4%), and was higher in urban (9·7% [95% CI, 9·4% - 10·1%]) than in rural (5·8% [95% CI, 5·7% - 6·0%]) areas. The three most prevalent morbidity combinations were hypertension with obesity (2·9% [95% CI, 2·8% - 3·1%]), hypertension with anemia (2·2% [95% CI, 2·1%– 2·3%]), and obesity with anemia (1·2% [95% CI, 1·1%– 1·2%]). The age-standardized multimorbidity prevalence varied from 3·4% (95% CI: 3·0% - 3·8%) in Chhattisgarh to 16·9% (95% CI: 13·2% - 21·5%) in Puducherry. Being a woman, being married, not currently smoking, greater household wealth, and living in urban areas were all associated with a higher risk of multimorbidity. Multimorbidity is common among young and middle-aged adults in India. This study can inform screening guidelines for chronic conditions and the targeting of relevant policies and interventions to those most in need.

## Introduction

Low-income and middle-income countries (LMICs) are facing a rapidly increasing disease burden from chronic diseases [1], largely due to population aging and changes in lifestyle [2, 3]. This epidemiological transition has been accompanied by rising levels of multimorbidity, which the World Health Organization (WHO) defined as the coexistence of two or more chronic conditions [4]. Multimorbidity is associated with high levels of healthcare service utilization and out-of-pocket expenditures [5, 6], high mortality [7–9], low quality of life [10], reduced functional status [7, 11], and high costs to the health system [12, 13]. Because healthcare financing and provision in LMICs is primarily focused on single diseases (e.g., HIV, malaria, or iron-deficiency anemia) through vertical health programs [14, 15], effectively dealing with this rise in multimorbidity will require fundamental and large-scale reforms that move towards a more horizontal and patient-centered healthcare delivery and financing system. Evidence on the epidemiology of multimorbidity in LMICs will be essential for guiding these efforts [16–18].

Multimorbidity in India is of particular global health importance as India’s population size accounts for more than one sixth of the world’s population [19], and because India has experienced an especially rapid epidemiological transition from acute infectious diseases to one predominated by chronic non-communicable conditions [20]. However, different states in India are in very different stages of this epidemiological transition [20]. As such, it is imperative for studies on multimorbidity in India to be sufficiently large and representative at state and district level to compare and contrast findings across and within states. While there have been several large studies on single chronic conditions [21–23], studies on multimorbidity in India have thus far been limited to samples in specific locales within certain states or small healthcare facility-based studies [9, 24, 25].

We conducted this analysis to address this important lack of evidence on occurence of multimorbidity and its associations by using nationally representative data on five of the most common chronic morbidities in India [20], namely anemia, asthma, diabetes, hypertension, and obesity. We also included HIV in our multimorbidity definition in a random subsample of participants that underwent an HIV test during the survey. These conditions constitute all chronic conditions that were measured in the most recent nationally representative health survey in India. Specifically, to inform the urgency with which health systems in different parts of this large and heterogeneous country need to transition from vertically organized, single-disease-focused healthcare delivery to a more horizontal approach with a focus on co-occurring chronic conditions, this study aimed to determine i) the prevalence of multimorbidity and specific chronic morbidity combinations at the national level in India, and ii) how the prevalence of multimorbidity varies among states and population subgroups within India.

## Methods

### Data sources

Because individual-level data from the fifth National Family Health Survey (NFHS) has not yet been made available, we analyzed data from the NFHS-4. The NFHS-4 is a household survey that was carried out between 2015 and 2016, and covered all states and union territories. The NFHS-4 used a two-stage cluster random sampling design (with district and rural versus urban location as strata), whereby primary sampling units (PSUs)–villages in rural areas and census enumeration blocks in urban areas–were selected with probability proportional to population size in the first stage. In the second stage, households within each PSU were selected through systematic random sampling, whereby the first household was selected randomly, and then every *x*^th^ household was sampled. Additional details on the sampling procedure are given in **S1 Text**. Owing to the survey’s focus on maternal and child health, the NFHS-4 sampled women aged 15–49 years in all selected households but sampled men aged 15 to 54 years in only a subsample of 15% of selected households. All men aged 15 to 54 years were sampled in these 15% of households, regardless of their relationship to any women sampled in the household. We only included individuals aged 15–49 years in our analysis to ensure comparability of gender estimates. An interviewer administered a questionnaire to all eligible individuals in the selected households. The response rate (for both the questionnaire and physical measurements detailed below) was 96·7% for women and 91·9% for men.

Before every interview, a respondent’s informed consent for participation in the survey was obtained. Special statements were included at both the beginning of the Household Questionnaire and the Individual Questionnaires. The statements explicitly explained the purpose of the survey. These statements also assured that all respondents were aware that participation in the survey is completely voluntary and that it is their right to refuse to answer any questions or stop the interview at any point [26].

### Ascertaining and defining morbidities

The NFHS-4 assessed five chronic morbidities: anemia, asthma, diabetes, hypertension, and obesity. In addition, a random subsample of 200,951 participants of all NFHS-4 participants were offered an HIV-test. Asthma was assessed through self-report, through a yes or no answer to the question “Do you currently have asthma?”. Other conditions used clinical or anthropometric measures in their derivation.

We defined anemia as a hemoglobin capillary blood concentration <11 g/dl, corresponding to moderate or severe anemia according to the 2011 WHO guidelines [27]. Prior to applying this cutoff, haemoglobin values were adjusted for smoking status (ascertained through self-report) and altitude (measured separately for each PSU with GPS devices) using formulas from the US Centers for Disease Control [28]. The NFHS-4 team measured hemoglobin using the HemoCueHb 201+ (HemoCue AB, Ängelholm, Sweden) and a capillary blood sample.

Diabetes was defined as having a raised blood glucose or having responded with ‘yes’ to at least one of “Do you currently have diabetes?” or “Have you sought treatment for this issue [diabetes]?”. The NFHS-team measured blood glucose using a handheld blood glucometer (FreeStyle Optium H [Abbott Laboratories, Abbott Park, USA]), whereby participants were not instructed to fast prior to the measurement. The capillary blood glucose measurement was converted to a plasma-equivalent value by multiplying with 1·11 [29]. We defined raised blood glucose as a plasma-equivalent glucose concentration ≥200 mg/dL (11·1 mmol/L) if not fasted, and ≥126 mg/dL (7·0 mmol/L) if fasted [30]. Participants were specifically asked about their fasting status. Fasting was defined as reporting no intake of food or drink, except plain water, for at least eight hours prior to the glucose sample being taken.

We defined hypertension as having a raised blood pressure (BP) or having responded with ‘yes’ to at least one of “Were you told on two or more different occasions by a doctor or other health professional that you had hypertension or high blood pressure?” or “To lower your blood pressure, are you now taking a prescribed medicine?”. We defined raised BP as having a mean systolic BP ≥140mmHg or a mean diastolic BP ≥90mmHg. The NFHS-4 team measured BP three times in the upper left arm with an electronic upper arm monitor (Omron HEM-8712 [Omron Corporation, Kyoto, Japan]), with at least five minutes between each measurement (and five minutes of quiet sitting prior to the first measurement). As is generally the standard in household surveys [31–33], we used only the last two measurements to compute mean systolic and diastolic BP.

Based on cutoffs specific to South Asia [34], we defined obesity as a Body Mass Index (BMI) ≥27·5 kg/m^2^. The NFHS-team measured weight using the SECA 874 U digital floor scale (seca GmbH, Hamburg, Germany) and height using the SECA 213 stadiometer (seca GmbH, Hamburg, Germany).

HIV was defined via an HIV blood test. A finger-prick blood specimen was taken among all participants (200,951 individuals) in a random subsample of households. All samples were first tested using an ELISA (enzyme-linked immunosorbent assay) test (Microlisa HIV, J. Mitra & Co. Pvt., New Delhi, India). Samples that tested positive, as well as a random sample of two percent of negative tests, were retested using a different ELISA test (SD Bioline HIV-1/2, Abbott Laboratories, Abbott Park, IL, USA). A positive result on both ELISA tests was recorded as HIV-positive. In the case of discordant results between the two ELISA tests, both ELISA tests were repeated in parallel. If the results still remained discordant, a Western Blot Test (Bio-Rad) was conducted at the National AIDS Research Institute (NARI) in Pune. The result of the Western Blot Test was then considered definitive.

### Sociodemographic variables

We examined how the prevalence of multimorbidity varied by the following sociodemographic variables asked in the survey questions: age, sex, education, household wealth quintile, marital status (currently married or not), current smoking, current consumption of smokeless tobacco, rural vs urban location, and state. Household wealth quintile was calculated separately for rural and urban locations using data on household ownership of 25 durable goods and seven key housing characteristics. Using the methodology developed by Filmer and Pritchett [35], we extracted the first component in a principal component analysis of these variables, and then divided this continuous asset index into quintiles. This is the standard approach used by all Demographic and Health Surveys [36]. More detail on the computation of the household wealth quintiles is provided in **S2 Text**. Additionally, as a measure of a district’s economic development, we also computed a district wealth quintile by calculating, separately for rural and urban areas, the median asset index in each district and then dividing districts into quintiles based on this value.

### Statistical analysis

Our analysis proceeded in four steps. First, we calculated national-level prevalence estimates for multimorbidity by age and rural vs urban areas, whereby all prevalence estimates in this manuscript used sampling weights that accounted for the survey design (including the higher probability of sampling women than men) and, unless prevalence was disaggregated by age, were age-standardized using the Global Burden of Disease Project’s age structure for India for 2015 [37]. We defined multimorbidity as having two or more of the five chronic conditions (anemia, asthma, diabetes, hypertension, and obesity) examined in this study. Second, we estimated the prevalence for each possible two- and three-morbidity combination among these five chronic morbidities. Third, we studied how the prevalence of multimorbidity varied among states by mapping prevalence by state. Fourth, to ascertain how the prevalence of multimorbidity varied by individual- and district-level characteristics, we regressed, separately for rural and urban areas, multimorbidity (as a binary variable) onto participants’ sociodemographic characteristics, district wealth quintile, and a random intercept for each district. We used Poisson regression models with a robust error structure, because it is a valid regression model for binary outcome data and yields a risk ratio (RR) [38], which is generally more easily interpreted than an Odds Ratio [39]. Standard errors were adjusted for clustering at the level of the PSU [40]. Fifth, to study patterns of multimorbidity among Indian adults living with HIV, we computed the prevalence of anemia, asthma, diabetes, hypertension, and obesity among those participants who had a positive HIV test. All analyses were complete case analyses and implemented in R (version 3.3.2; R Foundation).

### Ethics

This analysis received a determination of “not human subjects research” by the institutional review board of the Harvard T.H. Chan School of Public Health on 9 May 2018 because the authors had access to pseudonymized data only.

## Results

### Sample characteristics

749,119 individuals aged 15–49 years participated in the survey. 36,297 (4·8%) had a missing value for at least one morbidity, leaving 712,822 individuals (617,374 women and 95,448 men) for inclusion in the analysis (**S1 Fig**). The sample characteristics of those excluded from the analysis are shown in **S1 Table**. 26·5% (188,954 /712,822) of the analysis sample had no formal education, 68·8% (490,644/712,822) were married, 29·6% (210,798/712,822) were living in an urban area, and 5·4% (38,460/712,822) reported currently smoking (**Table 1**). 1·1% (8,067/712,822) of participants reported fasting at the time of the blood glucose measurement. Among those who underwent an HIV test, 0·23% (452/200,951) were HIV-positive. The sample characteristics among HIV-positive participants are shown in **S2 Table**.

**Table 1:** Sample characteristics^1^.

Characteristic	*Total*	*Women*	*Men*
n	712,822	617,374	95,448
Age Group, n (%)			
15–24 years	24,1854 (33·9)	209,193 (33·9)	32,661 (34·2)
25–34 years	21,1087 (29·6)	182,843 (29·6)	28,244 (29·6)
35–44 years	18,0555 (25·3)	156,456 (25·3)	24,099 (25·2)
45–49 years	79,326 (11·1)	68,882 (11·2)	10,444 (10·9)
*Missing*, *n (%)*	0 (0·0)	0 (0·0)	0 (0·0)
Education, n (%)			
No formal education	188,954 (26·5)	177,264 (28·7)	11,690 (12·2)
Some primary school	42,242 (5·9)	36,409 (5·9)	5,833 (6·1)
Completed primary school	47,709 (6·7)	41,750 (6·8)	5,959 (6·2)
Completed middle school	286,622 (40·2)	240,930 (39·0)	45,692 (47·9)
Completed secondary school	64,001 (9·0)	52,762 (8·5)	11,239 (11·8)
> Secondary school	83,294 (11·7)	68,259 (11·1)	15,035 (15·8)
*Missing*, *n (%)*	0 (0·0)	0 (0·0)	0 (0·0)
Household wealth quintile, n (%)			
Q1 (Poorest)	131,502 (18·4)	114873 (18·6)	16,629 (17·4)
Q2	141,413 (19·8)	122833 (19·9)	18,580 (19·5)
Q3	146,764 (20·6)	127152 (20·6)	19,612 (20·5)
Q4	144,701 (20·3)	124674 (20·2)	20,027 (21·0)
Q5 (Richest)	148,442 (20·8)	127842 (20·7)	20,600 (21·6)
*Missing*, *n (%)*	0 (0·0)	0 (0·0)	0 (0·0)
Currently married, n (%)	490,644 (68·8)	432960 (70·1)	57,684 (60·4)
*Missing*, *n (%)*	0 (0·0)	0 (0·0)	0 (0·0)
Urban area, n (%)	210,798 (29·6)	180802 (29·3)	29,996 (31·4)
*Missing*, *n (%)*	0 (0·0)	0 (0·0)	0 (0·0)
Smokes tobacco, n (%)	38,460 (5·4)	12930 (2·1)	25,530 (26·7)
*Missing*, *n (%)*	0 (0·0)	0 (0·0)	0 (0·0)
Uses smokeless tobacco, n (%)	86,730 (12·2)	55689 (9·0)	31,041 (32·5)
*Missing*, *n (%)*	0 (0·0)	0 (0·0)	0 (0·0)
Morbidity, n(%)			
Anemia	169,558 (23·8)	165187 (26·8)	4,371 (4·6)
Asthma	11,379 (1·6)	10308 (1·7)	1,071 (1·1)
Diabetes	18,979 (2·7)	15876 (2·6)	3,103 (3·3)
Hypertension	118,889 (16·7)	101461 (16·4)	17,428 (18·3)
Obesity	63,766 (8·9)	57212 (9·3)	6,554 (6·9)

Abbreviations: n = number; Q = quintile.

^1^ Sample characteristics are not weighted.

### Prevalence of multimorbidity at the national level

**Fig 1:**
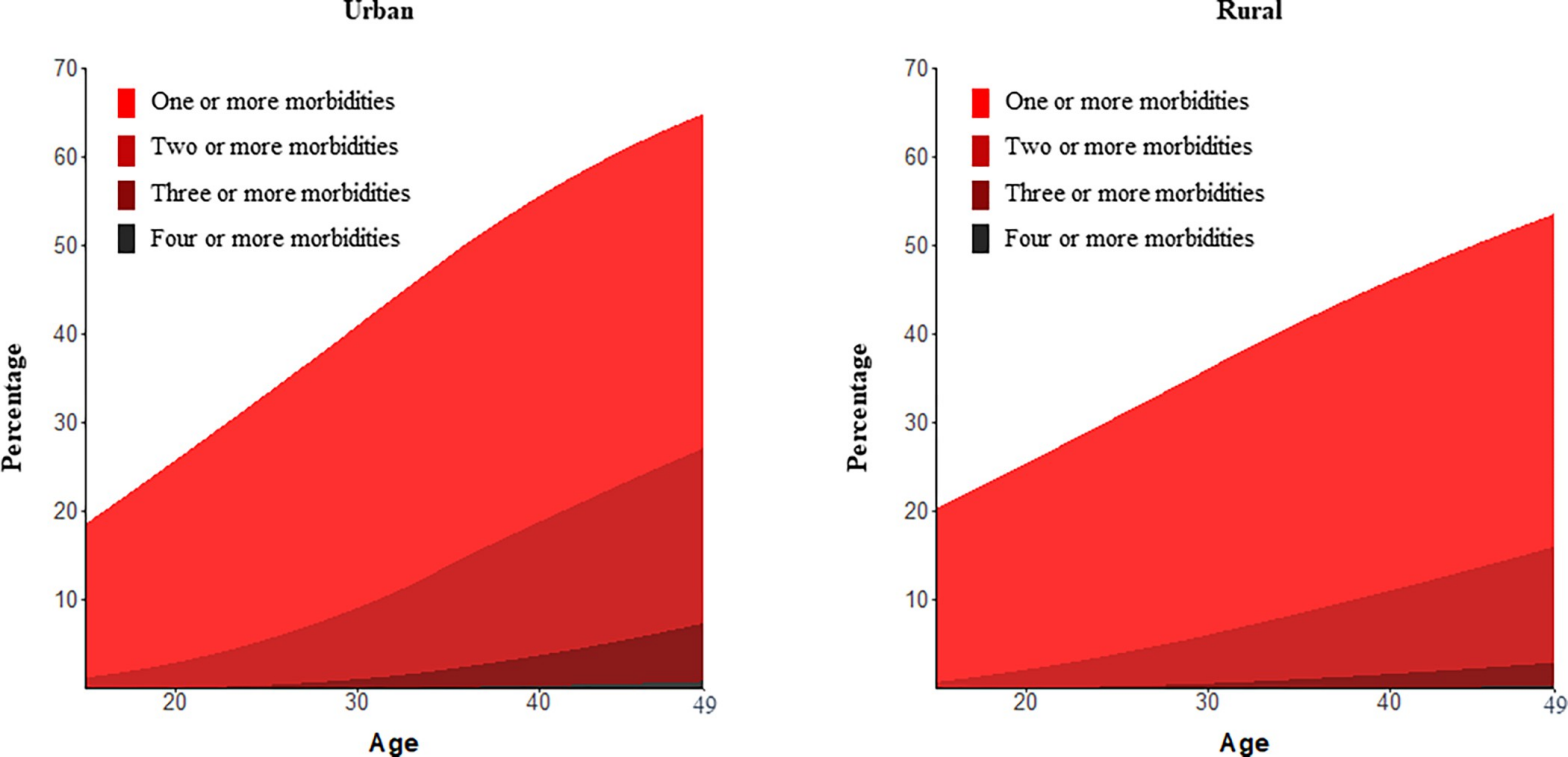
Prevalence of morbidity and multimorbidity by age and urbanicity in India^1^. ^1^ The prevalence of each number of morbidities (with 95% confidence intervals) is shown in **S3 Table**.

35·8% (95% CI, 35·4% - 36·1%) of participants had at least one of the five morbidities examined. At the national level, the prevalence of multimorbidity was 7·2% (95% CI, 7·1% - 7·4%). Multimorbidity prevalence was strongly associated with increasing age (**Fig 1** and **S3 Table**). Among those with at least one morbidity, 20·2% (95% CI, 19·9% - 20·6%) had two or more morbidities, 2·9% (95% CI, 2·8%– 3·1%) three or more morbidities, 0·3% (95% CI, 0·2%– 0·3%) four or five morbidities, and 0·01% (95% CI, 0·003%– 0·012%) five morbidities. The prevalence of multimorbidity was higher in urban than in rural areas (9·7% [95% CI, 9·4%– 10·1%] vs 5·8% [95% CI, 5·7%– 6·0%], respectively; p<0·001).

### National prevalence of different morbidity combinations

**Fig 2:**
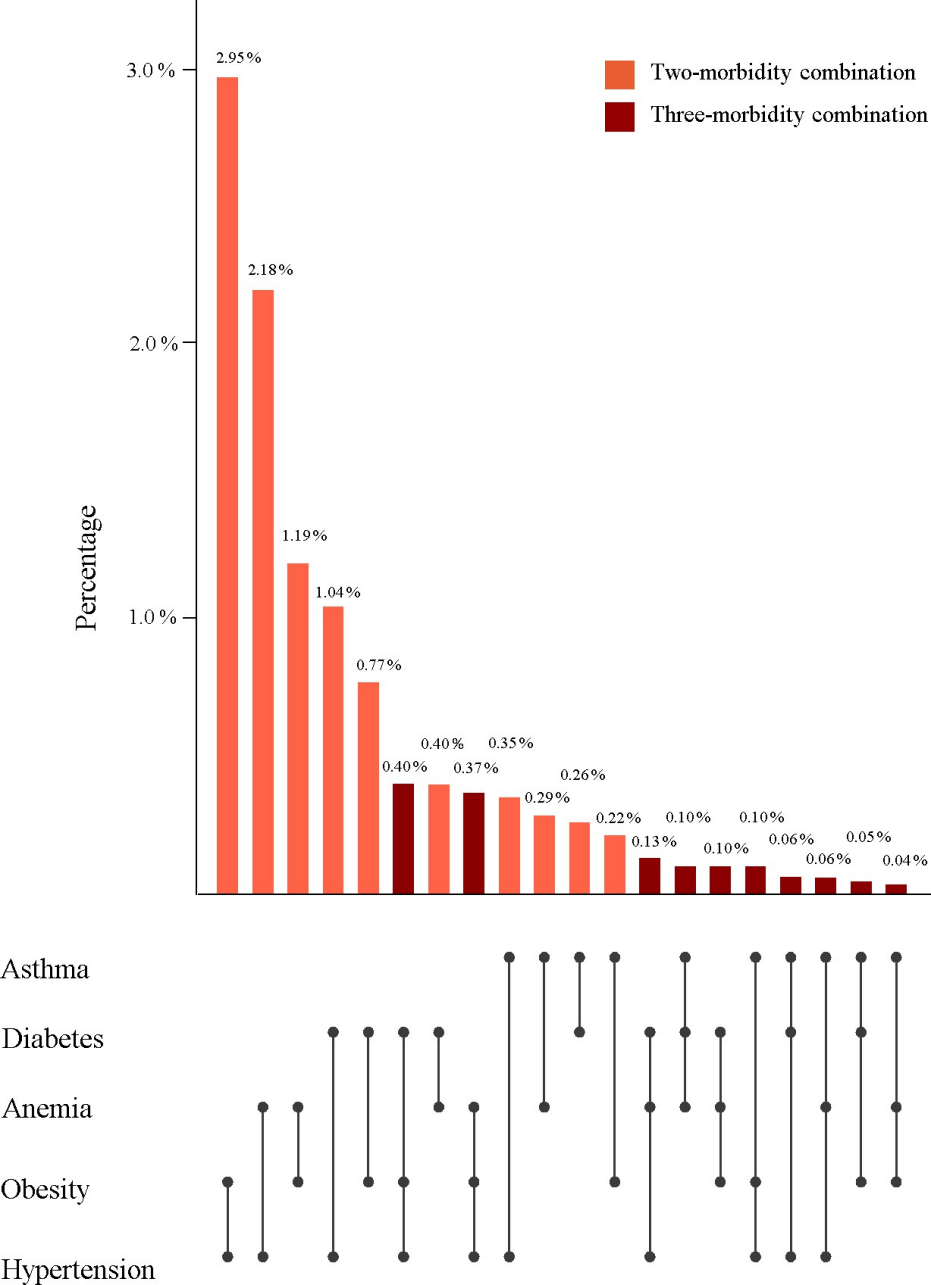
National prevalence of all two- and three-morbidity combinations^1^. ^1^ 95% confidence intervals can be found in S4 Table.

**Fig 2** shows the national prevalence of all possible combinations of two and three morbidities among the five morbidities studied. The two most common multimorbidity combinations both included hypertension: hypertension with obesity (2·9% [95% CI, 2·8%– 3·1%]), and hypertension with anemia (2·2% [95% CI, 2·1%– 2·3%]). The most common three-morbidity combination was diabetes with hypertension and obesity (0·4% [95% CI, 0·3% - 0·4%]).

### Variation of multimorbidity prevalence among states

**Fig 3:**
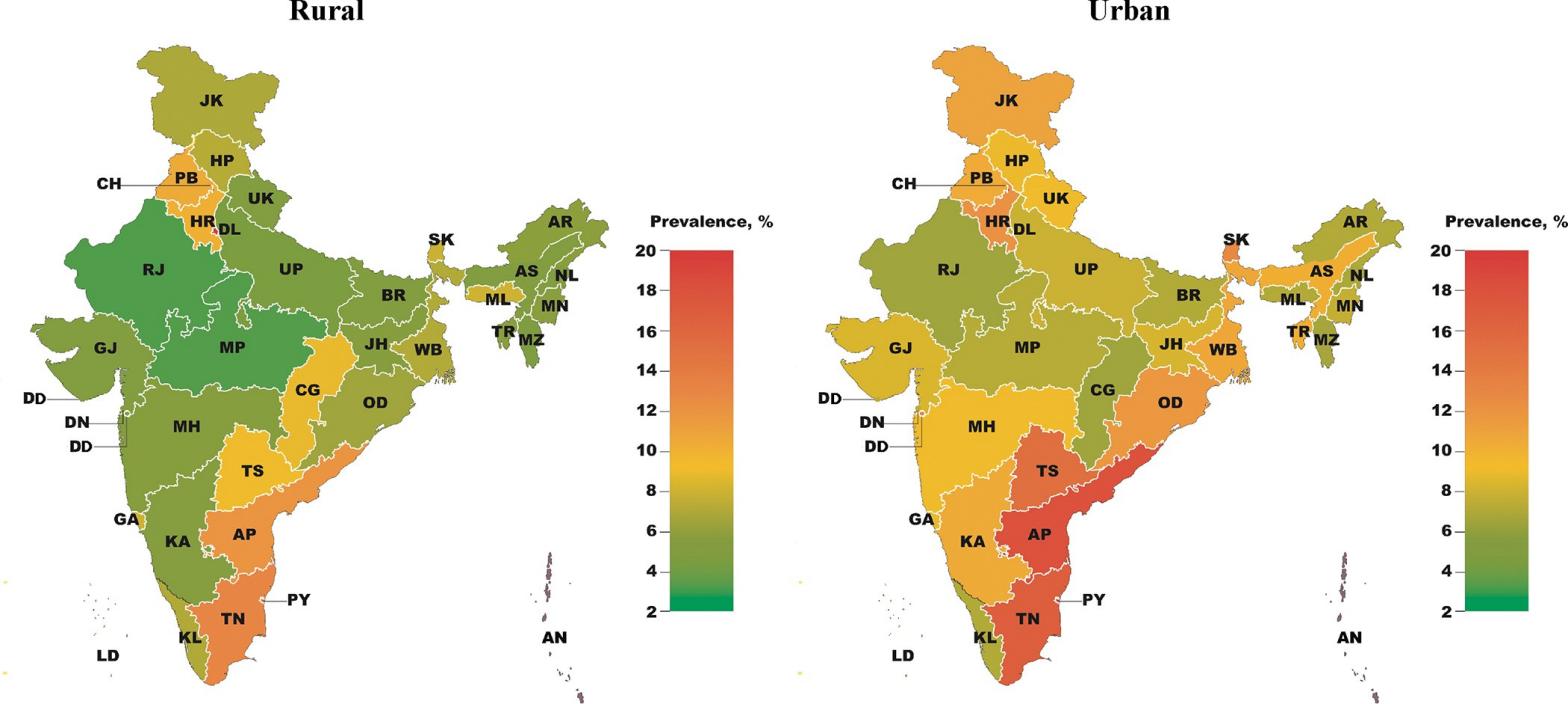
Age-standardized prevalence of multimorbidity by state and rural vs urban location^1,2,3^. ^1^ Point estimates and 95% CIs for each state and union territory can be found in **S5 Table**. ^2^ AN indicates Andaman and Nicobar Islands; AP, Andhra Pradesh; AR, Arunachal Pradesh; AS, Assam; BR, Bihar; CG, Chhattisgarh; CH, Chandigarh; DN, Dadra and Nagar Haveli; DL, Delhi; DD, Daman and Diu; GA, Goa; GJ, Gujarat; HR, Haryana; HP, Himachal Pradesh; JH, Jharkhand; JK, Jammu and Kashmir; KA, Karnataka; KL, Kerala; LD, Lakshadweep; MP, Madhya Pradesh; MH, Maharashtra; MN, Manipur; ML, Meghalaya; MZ, Mizoram; NL, Nagaland; OD, Odisha (Orissa); PB, Punjab; PY, Puducherry; RJ, Rajasthan; SK, Sikkim; TN, Tamil Nadu; TS, Telangana State; TR, Tripura; UP, Uttar Pradesh; UK, Uttarakhand (Uttaranchal); WB, West Bengal. ^3^ The map used for this figure was sourced from Survey of India, the national survey and mapping organization of India, Department of Science & Technology, Government of India.

Among states and union territories, the age-standardized prevalence of multimorbidity ranged from 3·4% (95% CI, 3·0% - 3·8%) in Chhattisgarh to 16·9% (95% CI, 13·2% - 21·5%) in Puducherry (**Fig 3** and **S5 Table**). Among states, multimorbidity prevalence was highest in urban areas of the South Indian states of Andhra Pradesh, Tamil Nadu, and Telangana, and lowest in rural areas of Madhya Pradesh and Rajasthan. The prevalence of multimorbidity was higher in urban areas than in rural areas in all Indian states except Meghalaya and Punjab. The states with the smallest absolute difference in multimorbidity prevalence between rural and urban areas were Punjab (10·0% [95% CI 8·9%– 11·2%] in rural areas vs 9·7% [95% CI 8·5%– 11·0%] in urban areas), Kerala (5·8% [95% CI 5·0%– 6·9%] in rural areas vs 6·3% [95% CI 5·3%– 7·5%] in urban areas), and Goa (7·1% [95% CI 5·0%– 9·9%] in rural areas vs 7·5% [95% CI 5·2%– 10·8%] in urban areas).

### Variation of multimorbidity prevalence by individual- and district-level characteristics

**Table 2** shows results from covariate-adjusted regression models. We find that i) women had a substantially higher risk of suffering from multimorbidity than men (RR of 1·95 [95% CI, 1·86–2·04] in rural and 1·76 [95% CI, 1·67–1·86] in urban areas); ii) increasing household wealth quintile was associated with multimorbidity in both rural and urban areas; and iii) smoking tobacco was negatively associated with multimorbidity. The median household wealth in a district was not associated with individuals’ risk of having a multimorbidity. We identified no consistent associations with educational attainment. Results from covariate-unadjusted regressions were similar (**S6 Table)**.

**Table 2:** Covariate-adjusted regressions of multimorbidity onto individuals’ sociodemographic characteristics and district wealth quintile^1^.

	Rural		Urban	
	*RR (95% CI)*	*P*	*RR (95% CI)*	*P*
Female	1·95 (1·86–2·04)	<0·001	1·76 (1·67–1·86)	<0·001
Age group				
15–24 years	1·00 (Ref.)		1·00 (Ref.)	
25–34 years	2·21 (2·12–2·30)	<0·001	2·65 (2·51–2·80)	<0·001
35–44 years	3·95 (3·79–4·11)	<0·001	5·05 (4·79–5·33)	<0·001
45–49 years	5·42 (5·19–5·66)	<0·001	6·84 (6·47–7·23)	<0·001
Household wealth quintile				
Q1 (Poorest)	1·00 (Ref.)		1·00 (Ref.)	
Q2	1·10 (1·06–1·15)	<0·001	1·31 (1·25–1·37)	<0·001
Q3	1·27 (1·22–1·32)	<0·001	1·52 (1·45–1·59)	<0·001
Q4	1·55 (1·48–1·61)	<0·001	1·64 (1·56–1·72)	<0·001
Q5 (Richest)	2·05 (1·96–2·14)	<0·001	1·72 (1·64–1·81)	<0·001
Education				
No formal education	1·00 (Ref.)		1·00 (Ref.)	
Some primary school	1·13 (1·08–1·18)	<0·001	1·08 (1·02–1·15)	0·011
Completed primary school	1·13 (1·08–1·17)	<0·001	1·10 (1·04–1·17)	<0·001
Completed middle school	1·09 (1·06–1·12)	<0·001	1·09 (1·05–1·13)	<0·001
Completed secondary school	1·08 (1·03–1·14)	0·001	1·01 (0·96–1·07)	0·607
> Secondary school	1·04 (0·99–1·09)	0·128	0·92 (0·87–0·96)	<0·001
Currently married	1·37 (1·32–1·42)	<0·001	1·30 (1·25–1·35)	<0·001
Currently smoking	0·89 (0·84–0·94)	<0·001	0·91 (0·84–0·97)	0·008
Currently using smokeless tobacco	1·05 (1·01–1·09)	0·006	1·03 (0·98–1·08)	0·220
District wealth quintile				
Q1 (Poorest)	1·00 (Ref.)		1·00 (Ref.)	
Q2	0·95 (0·88–1·03)	0·220	1·01 (0·93–1·10)	0·829
Q3	0·93 (0·85–1·01)	0·079	1·02 (0·94–1·12)	0·611
Q4	0·96 (0·88–1·05)	0·399	1·06 (0·97–1·16)	0·197
Q5 (Richest)	1·10 (1·00–1·22)	0·058	1·03 (0·93–1·13)	0·606

Abbreviations: RR = Risk Ratio; CI = Confidence Interval; Q = Quintile.

^1^ The regressions contained all variables shown in the table plus a random intercept for each district as independent variables.

### Subgroup analysis among HIV-positive participants

As was the case among the entire study population, the two most common comorbidities among the 469 participants with a positive HIV-test were hypertension (25·1%, 95% CI, 16·3% - 36·7%) and anemia (13·4%, 95% CI, 9·5% - 18·7%) (**S7 Table**). With a prevalence of 6·8% (95% CI, 1·7%– 23·2%), asthma was far more common among those with HIV than among the entire study population. The most prevalent three-morbidity combinations among HIV-positive participants were HIV with hypertension and obesity (7·6%, 95% CI, 2·2% - 22·9%) followed by HIV with asthma and obesity (4·8%, 95% CI, 0·7% - 26.5%).

## Discussion

This nationally representative study found a high prevalence of multimorbidity among young and middle-aged adults. This finding highlights the need to avoid single-disease-focused vertical programs in South Asia, which have been a mainstay of healthcare financing and delivery in LMICs. However, it is important to note that the shift away from single-disease-centered to person-centered care is more urgent for some states in India than others as we identified a vast degree of variation in the prevalence of multimorbidity between states, with urban areas in South India having a particularly high prevalence. Lastly, we found that being a woman, married, a non-smoker, living in a household with higher wealth, and living in urban areas were all associated with a higher risk of multimorbidity. This information is not only critical for targeting of appropriate interventions or prevention strategies to reach those most in need; they also imply that the prevalence of multimorbidity will rise in the future as India’s population ages and continues to undergo rapid economic development and urbanization [19, 41].

Preventing and effectively managing multimorbidity will require a shift towards a person- rather than disease- or episode-focused health system. Achieving continuity of person-centered care, across primary care and between primary and secondary care, is a major challenge for all health systems, but particularly so in India, which has multiple nationally managed vertical disease programs while much of primary and hospital care is managed by states [42]. To our knowledge, the National Programme for Prevention & Control of Cancer, Diabetes, Cardiovascular Diseases & Stroke (NPCDCS), which does not include anemia, asthma, and HIV, is the only nation-wide program in India that is focused on multiple related conditions. India also has a disproportionately large private care sector, in a setting where public spending on health care is one of the lowest worldwide [43, 44]. The private healthcare sector in India is highly fragmented and consists of a multitude of small independent providers with little to no coordination across providers [44]. The public healthcare system is also fragmented due to the presence of multiple disease-centered vertical programs, which operate in parallel to primary and secondary healthcare [42]. In addition, the majority of patient records are still paper-based and shared sporadically between healthcare facilities [45]. In addition to increased funding for primary care, the use of interdisciplinary professional care teams supported by integrated electronic care records and directed by clinical guidelines for managing common comorbidities could go a long way in transforming India’s health system to more effectively prevent and manage multimorbidity.

India has recently embarked on a major effort to develop integrated and comprehensive primary care services nationwide. Specifically, one of the two main components of India’s recently launched national health reform, *Ayushman Bharat* (Healthy India), is the establishment of 150,000 so-called health and wellness centers by 2022. In addition to maternal and child health services, these centers will place an emphasis on the prevention and treatment of chronic morbidities, particularly non-communicable diseases, such as diabetes and hypertension [46]. Our analysis can aid national health reforms in several ways. First, our study highlights in which states the need for care for multimorbidity is greatest, which can inform the geographic placement of health and wellness centers. Second, we show which morbidity combinations are most common, including among individuals with HIV, which can inform screening guidelines for healthcare workers at health and wellness centers and other healthcare facilities. Third, we determine which population groups are most likely to suffer from multimorbidity, which can inform not only screening guidelines but also the design of relevant interventions, such as health campaigns and community health worker interventions.

While studies in high-income settings tend to report a higher prevalence of multimorbidity among socioeconomically disadvantaged groups [47, 48], we found a positive association between household wealth and multimorbidity. However, our results do not suggest that multimorbidity is only a health problem among the wealthier strata of Indian society. For instance, the prevalence of multimorbidity in the lowest household wealth quintile was still considerable at 4·8% (95% CI, 4·6% - 5·1%). In addition, there was no clear association between these outcomes and educational attainment, which is another important indicator of socioeconomic status. Regardless, it may well be that the associations of multimorbidity with household wealth and education in India might become more similar to those seen in high-income settings as India continues to develop economically [49]. Another potentially surprising association in our regression analysis was the negative correlation between current smoking and multimorbidity. This association may be, among other potential reasons, a result of a negative association between smoking and obesity (which in turn is a risk factor for diabetes and hypertension) or of those diagnosed with a chronic morbidity being more likely to quit or underreport smoking.

This is by far the largest representative study of multimorbidity in South Asia to date. In fact, a recent systematic review on the prevalence of multimorbidity in South Asia identified 13 studies, of which only one–conducted among 320 adults in a rural area of one district–was carried out after 2010, and only three did not exclusively rely on self-report [24, 50]. The largest of these 13 studies had a sample size of 44,514 adults, was conducted in one neighborhood of Bangalore, and relied entirely on self-report to define morbidities [51]. Nonetheless, despite the limited literature on this subject, the overall body of evidence in LMICs suggests that the prevalence of multimorbidity is substantial in these settings [52].

This study has several limitations. First and foremost, while we were able to include many of the most important morbidities that are thought to affect India’s population [1, 53], the NFHS-4 did not assess an exhaustive list of these conditions. As such, the prevalence of multimorbidity in this analysis should not be interpreted as referring to the presence of two or more chronic conditions in general, but instead only has having two or more conditions of the five conditions that were assessed as part of the NFHS-4. Second, our dataset is only representative for individuals aged 15–49 years. However, given the country’s relatively young population structure, these age groups constituted an estimated 75·1% of India’s total population above the age of 15 years in 2015 [54]. Third, asthma, currently smoking, and consumption of smokeless tobacco were all defined through self-report only. We were unable to identify biomarker-defined smoking and smokeless tobacco consumption prevalence estimates for India. However, for asthma, our prevalence estimate of 1·5% (95% CI, 1·4% - 1·6%) was similar to the one obtained in a population-based study of 169,575 participants aged ≥15 years in 12 districts of India, which estimated a prevalence of 2·1% (no CI provided) using a detailed questionnaire on asthma symptoms [55]. Fourth, for those previously undiagnosed with diabetes, the definition of diabetes was based on a one-time capillary blood glucose measurement, which is insufficient for a clinical diagnosis of diabetes, especially since most participants were not fasted at the time of the measurement [56]. Fifth, men constituted merely 13·4% of participants in the NFHS-4. However, we used sampling weights to adjust for this higher probability of sampling men, and the absolute number of men included (95,448) was sufficiently high to obtain precise prevalence estimates for men. Lastly, HIV tests were conducted in a relatively small subgroup of 200,951 participants, leading to less precise multimorbidity prevalence estimates among participants living with HIV, which was the main reason for performing the HIV analysis separately.

India is facing a high prevalence of multimorbidity that may increase rapidly over the coming decades. Urgent reforms are needed to shift the health system’s focus away from episodic care for acute conditions towards longitudinal, integrated, and person-centered care. In addition, many of these chronic conditions share common risk factors–for example, air pollution is an important risk factor for hypertension and asthma [57–59], and poor diet quality is a risk factor for both anemia and obesity [60, 61]. Thus, population-based approaches targeting these underlying risk factors could have important effects. By providing the first detailed, nationally representative account of multimorbidity in India–including which states and population groups face the highest prevalence, and which morbidity combinations are most common–this study furnishes important evidence to provide both an impetus for future reforms, as well as inform the targeting of policies and interventions as part of India’s ongoing healthcare reform efforts.

## Supporting information

S1 Table Sample characteristics of excluded individuals^1^. (DOCX)

S2 Table Sample characteristics of individuals living with HIV. (DOCX)

S3 Table Age-multimorbidity association^1^. (DOCX)

S4 Table National prevalence of all two- and three-morbidity combinations. (DOCX)

S5 Table Multimorbidity prevalence per state. (DOCX)

S6 Table Covariate-unadjusted regressions: The association of sociodemographic characteristics with multimorbidity. (DOCX)

S7 Table Prevalence of different morbidity combinations among individuals with HIV^1^. (DOCX)

S1 Text Detailed description of sampling procedure. (DOCX)

S2 Text Computation of household wealth quintiles. (DOCX)

S1 Fig Flowchart of HIV testing. (DOCX)
