# Supplementary material for: Patterns of multimorbidity in India: A nationally representative cross-sectional study of individuals aged 15 to 49 years
Source: PLOS Glob Public Health. 2022 Aug 17;2(8):e0000587. doi: 10.1371/journal.pgph.0000587 (PMC10021201; doi:10.1371/journal.pgph.0000587)
Supplement: S1 Table — (DOCX) [file pgph.0000587.s001.docx]

# S1 Table. Sample characteristics of excluded individuals^1^

| **Characteristic** | **Excluded individuals** | | |
| --- | --- | --- | --- |
|  | ***Total*** | ***Women*** | ***Men*** |
| n | 36297 | 30077 | 6220 |
| Age Group, n (%) |  |  |  |
| 15-24 years | 13446 (37.0) | 11215 (37.3) | 2231 (35.9) |
| 25-34 years | 10447 (28.8) | 8544 (28.4) | 1903 (30.6) |
| 35-44 years | 8533 (23.5) | 7091 (23.6) | 1442 (23.2) |
| 45-54 years | 3871 (10.7) | 3227 (10.7) | 644 (10.4) |
| Education, n (%) |  |  |  |
| No formal education | 8352 (23.0) | 7601 (25.3) | 751 (12.1) |
| <Primary School | 2190 ( 6.0) | 1802 ( 6.0) | 388 ( 6.2) |
| Primary School | 1990 ( 5.5) | 1654 ( 5.5) | 336 ( 5.4) |
| Middle School | 15076 (41.5) | 12137 (40.4) | 2939 (47.3) |
| Secondary School | 3436 ( 9.5) | 2733 ( 9.1) | 703 (11.3) |
| >Secondary School | 5253 (14.5) | 4150 (13.8) | 1103 (17.7) |
| Household wealth quintile, n (%) |  |  |  |
| Q1 (Poorest) | 6478 (17.8) | 5437 (18.1) | 1041 (16.7) |
| Q2 | 7105 (19.6) | 5882 (19.6) | 1223 (19.7) |
| Q3 | 7539 (20.8) | 6277 (20.9) | 1262 (20.3) |
| Q4 | 7346 (20.2) | 6047 (20.1) | 1299 (20.9) |
| Q5 (Richest) | 7829 (21.6) | 6434 (21.4) | 1395 (22.4) |
| Currently married, n (%) | 22113 (60.9) | 18730 (62.3) | 3383 (54.4) |
| Urban area, n (%) | 13017 (35.9) | 10680 (35.5) | 2337 (37.6) |
| Tobacco consumption, n (%) |  |  |  |
| smokes Tobacco | 2064 ( 5.7) | 477 ( 1.6) | 1587 (25.5) |
| uses smokeless tobacco | 4968 (13.7) | 3020 (10.0) | 1948 (31.3) |
| Morbidity, n(%) |  |  |  |
| Diabetes | 533 ( 1.5) | 432 ( 1.4) | 101 ( 1.6) |
| Hypertension | 1531 ( 4.2) | 1295 ( 4.3) | 236 ( 3.8) |
| Obesity | 1872 ( 5.2) | 1656 ( 5.5) | 216 ( 3.5) |
| Asthma | 455 ( 1.3) | 397 ( 1.3) | 58 ( 0.9) |
| Anemia | 4228 (11.6) | 4089 (13.6) | 139 ( 2.2) |

Abbreviations: n=number; Q=quintile.

^1^ Sample characteristics are not weighted.
