# Supplementary material for: Patterns of multimorbidity in India: A nationally representative cross-sectional study of individuals aged 15 to 49 years
Source: PLOS Glob Public Health. 2022 Aug 17;2(8):e0000587. doi: 10.1371/journal.pgph.0000587 (PMC10021201; doi:10.1371/journal.pgph.0000587)
Supplement: S2 Table — (DOCX) [file pgph.0000587.s002.docx]

# S2 Table. Sample characteristics of individuals living with HIV

| **Characteristic** | **Excluded individuals** | | |
| --- | --- | --- | --- |
|  | ***Total*** | ***Women*** | ***Men*** |
| n | 452 | 237 | 215 |
| Age Group, n (%) |  |  |  |
| 15-24 years | 73 (16.2) | 32 (13.5) | 41 (19.1) |
| 25-34 years | 152 (33.6) | 88 (37.1) | 64 (29.8) |
| 35-44 years | 163 (36.1) | 88 (37.1) | 75 (34.9) |
| 45-54 years | 64 (14.2) | 29 (12.2) | 35 (16.3) |
| Education, n (%) |  |  |  |
| No formal education | 91 (20.1) | 57 (24.1) | 34 (15.8) |
| <Primary School | 43 ( 9.5) | 19 ( 8.0) | 24 (11.2) |
| Primary School | 27 ( 6.0) | 17 ( 7.2) | 10 ( 4.7) |
| Middle School | 213 (47.1) | 115 (48.5) | 98 (45.6) |
| Secondary School | 36 ( 8.0) | 13 ( 5.5) | 23 (10.7) |
| >Secondary School | 42 ( 9.3) | 16 ( 6.8) | 26 (12.1) |
| Household wealth quintile, n (%) |  |  |  |
| Q1 (Poorest) | 87 (19.2) | 51 (21.5) | 36 (16.7) |
| Q2 | 88 (19.5) | 47 (19.8) | 41 (19.1) |
| Q3 | 108 (23.9) | 50 (21.1) | 58 (27.0) |
| Q4 | 89 (19.7) | 46 (19.4) | 43 (20.0) |
| Q5 (Richest) | 80 (17.7) | 43 (18.1) | 37 (17.2) |
| Currently married, n (%) | 293 (64.8) | 144 (60.8) | 149 (69.3) |
| Urban area, n (%) | 210 (46.5) | 110 (46.4) | 100 (46.5) |
| Tobacco consumption, n (%) |  |  |  |
| smokes Tobacco | 86 (19.0) | 12 ( 5.1) | 74 (34.4) |
| uses smokeless tobacco | 156 (34.5) | 60 (25.3) | 96 (44.7) |
| Morbidity, n(%) |  |  |  |
| Diabetes | 18 ( 4.0) | 8 ( 3.4) | 10 ( 4.7) |
| Hypertension | 113 (25.0) | 57 (24.1) | 56 (26.0) |
| Obesity | 21 ( 4.6) | 14 ( 5.9) | 7 ( 3.3) |
| Asthma | 9 ( 2.0) | 4 ( 1.7) | 5 ( 2.3) |
| Anemia | 99 (21.9) | 83 (35.0) | 16 ( 7.4) |
