# Supplementary material for: Patterns of multimorbidity in India: A nationally representative cross-sectional study of individuals aged 15 to 49 years
Source: PLOS Glob Public Health. 2022 Aug 17;2(8):e0000587. doi: 10.1371/journal.pgph.0000587 (PMC10021201; doi:10.1371/journal.pgph.0000587)
Supplement: S3 Table — (DOCX) [file pgph.0000587.s003.docx]

# S3 Table. Age-Multimorbidity association^1^

| Age | Zero morbidities | One morbidity | Two morbidities | Three morbidities |
| --- | --- | --- | --- | --- |
| 15-25 | 76.59 (76.18-76.99) | 23.41 (23.01-23.82) | 1.55 (1.46-1.66) | 0.06 (0.04-0.08) |
| 26-35 | 66.63 (66.12-67.13) | 33.37 (32.87-33.88) | 3.80 (3.64-3.97) | 0.21 (0.17-0.25) |
| 36-45 | 55.09 (54.50-55.68) | 44.91 (44.32-45.50) | 8.00 (7.70-8.31) | 0.62 (0.55-0.70) |
| 46-49 | 48.78 (47.81-49.75) | 51.22 (50.25-52.19) | 11.45 (10.88-12.04) | 1.27 (1.07-1.50) |

^1^The percentage of individuals with zero morbities, one morbidity, two morbities and three morbities were calculated per age group using the sampling weights applied to all national indicators.
