# Supplementary material for: Patterns of multimorbidity in India: A nationally representative cross-sectional study of individuals aged 15 to 49 years
Source: PLOS Glob Public Health. 2022 Aug 17;2(8):e0000587. doi: 10.1371/journal.pgph.0000587 (PMC10021201; doi:10.1371/journal.pgph.0000587)
Supplement: S4 Table — (DOCX) [file pgph.0000587.s004.docx]

**S4 Table. National prevalence of all two- and three-morbidity combinations**

| **Morbidity combination** | **Point estimate** | **Low CI** | **High CI** |
| --- | --- | --- | --- |
| Hypertension-Obesity | 2.95 | 2.84 | 3.07 |
| Asthma-Obesity | 0.22 | 0.19 | 0.24 |
| Anemia-Obesity | 1.19 | 1.15 | 1.24 |
| Diabetes-Obesity | 0.77 | 0.72 | 0.82 |
| Anemia-Diabetes | 0.40 | 0.37 | 0.43 |
| Anemia-Asthma | 0.29 | 0.26 | 0.31 |
| Anemia-Hypertension | 2.18 | 2.11 | 2.25 |
| Diabetes-Asthma | 0.26 | 0.21 | 0.32 |
| Diabetes-Hypertension | 1.04 | 0.98 | 1.10 |
| Asthma-Hypertension | 0.35 | 0.32 | 0.38 |
| Diabetes-Hypertension-Obesity | 0.40 | 0.37 | 0.43 |
| Diabetes-Hypertension-Asthma | 0.06 | 0.05 | 0.08 |
| Diabetes-Hypertension-Anemia | 0.13 | 0.12 | 0.15 |
| Diabetes-Obesity-Asthma | 0.05 | 0.04 | 0.06 |
| Diabetes-Obesity-Anemia | 0.10 | 0.09 | 0.12 |
| Diabetes-Asthma-Anemia | 0.10 | 0.09 | 0.12 |
| Hypertension-Obesity-Asthma | 0.10 | 0.08 | 0.12 |
| Hypertension-Obesity-Anemia | 0.37 | 0.35 | 0.39 |
| Hypertension-Asthma-Anemia | 0.06 | 0.05 | 0.07 |
| Obesity-Asthma-Anemia | 0.04 | 0.03 | 0.04 |
