# Supplementary material for: Patterns of multimorbidity in India: A nationally representative cross-sectional study of individuals aged 15 to 49 years
Source: PLOS Glob Public Health. 2022 Aug 17;2(8):e0000587. doi: 10.1371/journal.pgph.0000587 (PMC10021201; doi:10.1371/journal.pgph.0000587)
Supplement: S5 Table — (DOCX) [file pgph.0000587.s005.docx]

# S5 Table. Multimorbidity prevalence per state

| **State** | **Prevalence of multimorbidity** | **Low CI** | **High CI** |
| --- | --- | --- | --- |
| Andaman and Nicobar Islands | 16.3 | 14.1 | 18.7 |
| Andhra Pradesh | 13.3 | 12.2 | 14.5 |
| Arunachal Pradesh | 5.6 | 4.9 | 6.4 |
| Assam | 5.5 | 5.0 | 6.0 |
| Bihar | 5.1 | 4.7 | 5.6 |
| Chandigarh | 12.4 | 8.8 | 17.0 |
| Chhattisgarh | 3.4 | 3.0 | 3.8 |
| Dadra and Nagar Haveli | 8.9 | 6.2 | 12.5 |
| Daman and Diu | 6.7 | 4.1 | 10.6 |
| Delhi | 7.6 | 6.3 | 9.1 |
| Goa | 7.4 | 5.7 | 9.5 |
| Gujarat | 6.3 | 5.8 | 6.9 |
| Haryana | 10.8 | 9.9 | 11.9 |
| Himachal Pradesh | 6.9 | 6.2 | 7.8 |
| Jammu and Kashmir | 7.4 | 6.7 | 8.2 |
| Jharkhand | 6.0 | 5.5 | 6.5 |
| Karnataka | 7.1 | 6.4 | 7.8 |
| Kerala | 6.1 | 5.4 | 6.8 |
| Lakshadweep | 9.0 | 6.4 | 12.5 |
| Madhya Pradesh | 4.4 | 4.2 | 4.7 |
| Maharashtra | 6.9 | 6.2 | 7.6 |
| Manipur | 6.2 | 5.6 | 6.9 |
| Meghalaya | 7.2 | 5.9 | 8.9 |
| Mizoram | 5.8 | 5.0 | 6.7 |
| Nagaland | 5.2 | 4.5 | 6.0 |
| Odisha | 7.1 | 6.6 | 7.7 |
| Puducherry | 16.9 | 13.2 | 21.5 |
| Punjab | 9.9 | 9.1 | 10.8 |
| Rajasthan | 3.9 | 3.6 | 4.2 |
| Sikkim | 9.2 | 8.0 | 10.7 |
| Tamil Nadu | 14.4 | 13.4 | 15.4 |
| Telangana | 11.6 | 10.4 | 12.9 |
| Tripura | 6.9 | 5.9 | 8.0 |
| Uttar Pradesh | 5.1 | 4.8 | 5.4 |
| Uttarakhand | 6.0 | 5.4 | 6.7 |
| West Bengal | 7.7 | 7.0 | 8.5 |
