# Supplementary material for: Patterns of multimorbidity in India: A nationally representative cross-sectional study of individuals aged 15 to 49 years
Source: PLOS Glob Public Health. 2022 Aug 17;2(8):e0000587. doi: 10.1371/journal.pgph.0000587 (PMC10021201; doi:10.1371/journal.pgph.0000587)
Supplement: S6 Table — (DOCX) [file pgph.0000587.s006.docx]

# S6 Table. Covariate-unadjusted regressions: The association of sociodemographic characteristics with multimorbidity

|  | **Rural** | | **Urban** | |
| --- | --- | --- | --- | --- |
|  | *RR (95% CI)* | *P* | *RR (95% CI)* | *P* |
| Female | 1.90 (1.82-1.98) | <0.001 | 1.82 (1.73-1.91) | <0.001 |
| Age group |  |  |  |  |
| 15 – 24 years | 1.00 (Ref.) |  | 1.00 (Ref.) |  |
| 25 – 34 years | 2.67 (2.57-2.77) | <0.001 | 3.17 (3.02-3.33) | <0.001 |
| 35 – 44 years | 4.72 (4.56-4.90) | <0.001 | 6.31 (6.02-6.62) | <0.001 |
| 45 – 54 years | 6.43 (6.18-6.68) | <0.001 | 8.63 (8.22-9.07) | <0.001 |
| Household wealth quintile |  |  |  |  |
| Q1 (Poorest) | 1.00 (Ref.) |  | 1.00 (Ref.) |  |
| Q2 | 1.07 (1.03-1.12) | 0.001 | 1.39 (1.33-1.45) | <0.001 |
| Q3 | 1.27 (1.22-1.32) | <0.001 | 1.64 (1.57-1.72) | <0.001 |
| Q4 | 1.64 (1.57-1.71) | <0.001 | 1.76 (1.68-1.84) | <0.001 |
| Q5 (Richest) | 2.37 (2.28-2.47) | <0.001 | 1.85 (1.77-1.94) | <0.001 |
| Education |  |  |  |  |
| No formal education | 1.00 (Ref.) |  | 1.00 (Ref.) |  |
| Some primary school | 1.00 (0.96-1.04) | 0.825 | 0.94 (0.89-1.00) | 0.043 |
| Completed primary school | 1.02 (0.98-1.06) | 0.430 | 1.01 (0.96-1.06) | 0.732 |
| Completed middle school | 0.76 (0.74-0.78) | <0.001 | 0.79 (0.77-0.82) | <0.001 |
| Completed secondary school | 0.75 (0.72-0.79) | <0.001 | 0.73 (0.70-0.77) | <0.001 |
| > Secondary school | 0.74 (0.71-0.77) | <0.001 | 0.70 (0.67-0.73) | <0.001 |
| Currently married | 2.74 (2.66-2.82) | <0.001 | 3.08 (2.97-3.19) | <0.001 |
| Tobacco consumption |  |  |  |  |
| Smoking | 0.76 (0.72-0.80) | <0.001 | 0.70 (0.66-0.75) | <0.001 |
| Smokeless | 0.95(0.92-0.98) | 0.003 | 0.87 (0.83-0.91) | <0.001 |
| District wealth quintile |  |  |  |  |
| Q1 (Poorest) | 1.00 (Ref.) |  | 1.00 (Ref.) |  |
| Q2 | 0.98 (0.94-1.02) | 0.253 | 1.06 (1.00-1.12) | 0.036 |
| Q3 | 1.19 (1.14-1.25) | <0.001 | 1.23 (1.17-1.31) | <0.001 |
| Q4 | 0.99 (0.95-1.04) | 0.739 | 1.05 (0.99-1.11) | 0.101 |
| Q5 (Richest) | 1.48 (1.42-1.54) | <0.001 | 1.12 (1.06-1.18) | <0.001 |

Abbreviations: RR = Risk Ratio; CI = Confidence Interval; Q = Quintile.
