# Supplementary material for: Patterns of multimorbidity in India: A nationally representative cross-sectional study of individuals aged 15 to 49 years
Source: PLOS Glob Public Health. 2022 Aug 17;2(8):e0000587. doi: 10.1371/journal.pgph.0000587 (PMC10021201; doi:10.1371/journal.pgph.0000587)
Supplement: S7 Table — (DOCX) [file pgph.0000587.s007.docx]

# S7 Table. Prevalence of different morbidity combinations among individuals with HIV^1^

| **Morbidity combination** | **Point estimate** | **Low CI** | **High CI** |
| --- | --- | --- | --- |
| Hypertension-HIV | 25.1 | 16.3 | 36.7 |
| Obesity-HIV | 8.5 | 2.8 | 22.7 |
| Diabetes-HIV | 3.7 | 1.5 | 8.7 |
| Asthma-HIV | 6.8 | 1.7 | 23.2 |
| Anemia-HIV | 13.4 | 9.5 | 18.7 |
| Hypertension-Obesity-HIV | 7.6 | 2.2 | 22.9 |
| Asthma-Obesity-HIV | 4.8 | 0.7 | 26.5 |
| Anemia-Obesity-HIV | 1.6 | 0.5 | 5.6 |
| Diabetes-Obesity-HIV | 0.2 | 0.0 | 1.2 |
| Anemia-Diabetes-HIV | 0.4 | 0.1 | 1.8 |
| Anemia-Asthma-HIV | 0.6 | 0.1 | 3.4 |
| Anemia-Hypertension-HIV | 3.7 | 1.7 | 7.9 |
| Diabetes-Hypertension-HIV | 1.4 | 0.4 | 4.9 |
| Asthma-Hypertension-HIV | 4.8 | 0.7 | 26.5 |
| Diabetes-Hypertension-Obesity-HIV | 0.2 | 0.01 | 1.2 |
| Diabetes-Hypertension-Anemia-HIV | 0.1 | 0.01 | 0.5 |
| Hypertension-Obesity-Asthma-HIV | 4.8 | 0.7 | 26.5 |
| Hypertension-Obesity-Anemia-HIV | 1.3 | 0.3 | 5.9 |

^1^ All combinations not included in this table had a point estimate lower than 0.0%.
