# Supplementary material for: Patterns of multimorbidity in India: A nationally representative cross-sectional study of individuals aged 15 to 49 years
Source: PLOS Glob Public Health. 2022 Aug 17;2(8):e0000587. doi: 10.1371/journal.pgph.0000587 (PMC10021201; doi:10.1371/journal.pgph.0000587)
Supplement: S1 Text — (DOCX) [file pgph.0000587.s008.docx]

# S1 Text. Detailed description of sampling procedure

In the NFHS-4, a stratified two-stage random sampling design was used with primary sampling units (PSUs) defined as villages in rural areas and Census Enumeration Blocks (CEBs) in urban areas according to the 2011 Census for India. Small PSUs with fewer than 40 households were included in the nearest PSU. Strata were rural/urban areas within each of the 640 districts of India. The villages in each rural stratum and CEBs in each urban stratum were chosen using the probability proportional to population size (PPS) method. PSUs larger than 300 estimated households were subdivided into two segments of 100-150 households, of which two were randomly selected (again, using PPS) for the survey. In each of these clusters (PSUs or PSU segments), 22 households were randomly chosen by systematic random sampling. Four Computer Assisted Personal Interviewing (CAPI) questionnaires (Household Questionnaire, Woman’s Questionnaire, Man’s Questionnaire, and Biomarker Questionnaire) were administered in each household. The Household Questionnaire listed all usual members of the household and in addition all visitors who had stayed in the household the night before the interview. Basic demographic information including age, sex, schooling, marital status, relationship to the head of the household was documented for each listed person. Personal interviews using the Woman's and Man's Questionnaire as well as physical measurements using the Biomarker Questionnaire were carried out among all listed persons of age 15-49 years (Women) or 15-54 years (Men). The Woman's Questionnaire, Man's Questionnaire and the Biomarker Questionnaire contained all key measures analyzed in this publication. More detailed information is publically available at [www.dhsprogram.com](http://www.dhsprogram.com) and [www.rchiips.org/NFHS/about.shtml](http://www.rchiips.org/NFHS/about.shtml).
