# Supplementary material for: Patterns of multimorbidity in India: A nationally representative cross-sectional study of individuals aged 15 to 49 years
Source: PLOS Glob Public Health. 2022 Aug 17;2(8):e0000587. doi: 10.1371/journal.pgph.0000587 (PMC10021201; doi:10.1371/journal.pgph.0000587)
Supplement: S2 Text — (DOCX) [file pgph.0000587.s009.docx]

# S2 Text. Computation of household wealth quintiles

The household wealth quintiles were calculated and provided by the DHS based on the following key dwelling characteristics:

- Main roof material
- Main wall material
- Main material of floor
- Type of cooking fuel
- Type of toilet facility
- Source of drinking water

Ownership of the following durable goods (‘assets’) was also used for the estimation:

| - Mattress |
| --- |
| - Pressure cooker |
| - Chair |
| - Cot or bed |
| - Table |
| - Electric fan |
| - Radio or transistor |
| - Black and white television |
| - Colour television |
| - Sewing machine |
| - Mobile telephone |
| - Telephone (non-mobile) |
| - Internet |
| - Computer |
| - Refrigerator |
| - Air conditioner/cooler |
| - Washing machine |
| - Watch or clock |
| - Bicycle |
| - Motorcycle or Scooter |
| - Animal-drawn cart |
| - Car |
| - Water pump |
| - Thresher |
| - Tractor |

The analysis was performed in a three step process: First, The above listed items were encoded as a binary indicator. Second, a Principal Component Analysis (PCA) was run separately for rural and urban areas with the first (unrotated) component being selected. The resulting standardized score after this step has by definition a standard deviation of one, a mean of zero, and wealthier households receive higher scores. The variable was then divided into quintiles in a third step to improve interpretability of the resulting index. A detailed description of the analysis process used has been published.^1,2^
