# Supplementary figures and images for: Patterns of multimorbidity in India: A nationally representative cross-sectional study of individuals aged 15 to 49 years
Source: PLOS Glob Public Health. 2022 Aug 17;2(8):e0000587. doi: 10.1371/journal.pgph.0000587 (PMC10021201; doi:10.1371/journal.pgph.0000587)

# S1 Fig. Flowchart of HIV testing


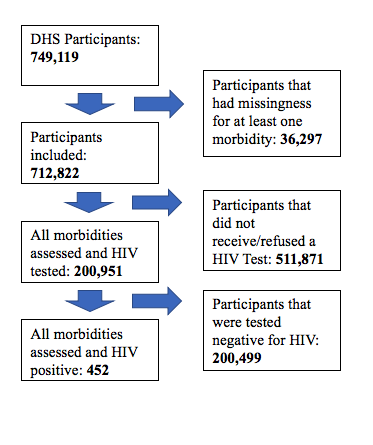

Supplement: S1 Fig — (DOCX) [file pgph.0000587.s010.docx]
